# Cooling‐induced cutaneous vasodilatation is mediated by small‐conductance, calcium‐activated potassium channels in tail arteries from male mice

**DOI:** 10.14814/phy2.15884

**Published:** 2023-11-27

**Authors:** Fumin Chang, Sheila Flavahan, Nicholas A. Flavahan

**Affiliations:** ^1^ Department of Anesthesiology Johns Hopkins University Baltimore Maryland USA

**Keywords:** cutaneous arteries, myoendothelial coupling, Raynaud's phenomenon, thermoregulation, vasodilatation

## Abstract

Cooling causes cutaneous dilatation to restrain cold‐induced constriction and prevent tissue injury. Cooling increases communication through myoendothelial gap junctions (MEGJs), thereby increasing endothelium‐derived hyperpolarization (EDH)‐type dilatation. EDH is initiated by calcium‐activated potassium channels (K_Ca_) activated by endothelial stimuli or muscle‐derived mediators traversing MEGJs (myoendothelial feedback). The goal of this study was to determine the individual roles of K_Ca_ with small (SK3) and intermediate (IK1) conductance in cooling‐induced dilatation. Vasomotor responses of mice isolated cutaneous tail arteries were analyzed by pressure myography at 37°C and 28°C. Cooling increased acetylcholine‐induced EDH‐type dilatation during inhibition of NO and prostacyclin production. IK1 inhibition did not affect dilatations to acetylcholine, whereas SK3 inhibition inhibited dilatation at both temperatures. Cooling uncovered myoendothelial feedback to inhibit constrictions in U46619. IK1 inhibition did not affect U46619 constrictions, whereas SK3 inhibition abolished the inhibitory effect of cooling without affecting U46619 constriction at 37°C. Immunoblots confirmed SK3 expression, which was localized (immunofluorescence) to holes in the internal elastic lamina consistent with myoendothelial projections. Immunoblots and Immunofluorescence did not detect IK1. Studies in non‐cutaneous arteries have highlighted the predominant role of IK1 in EDH‐type dilatation. Cutaneous arteries are distinctly reliant on SK3, which may enable EDH‐type dilation to be amplified by cooling.

## 1 INTRODUCTION

The cutaneous vascular system has unique structural and functional features that enable it to contribute to thermoregulation and the maintenance of core temperature (Flavahan, 2015a). Cold exposure causes cutaneous vasoconstriction that acts to restrict heat loss to the environment. The cold‐induced constriction is mediated by a reflex increase in sympathetic adrenergic outflow and a direct effect of local cooling to amplify constriction to sympathetic neurotransmission (Flavahan, 2015a). Indeed, local cooling selectively increases the constrictor activity of smooth muscle α2‐ARs (Chotani et al., 2000; Flavahan et al., 1985). Unlike smooth muscle cells from other circulations, which rely predominantly on α1‐adrenoceptors (α1‐ARs), cutaneous smooth muscle cells have much higher expression and activity of α2‐ARs (Chotani et al., 2004; Flavahan, 2005; Flavahan et al., 1985). The increased prominence of α2‐ARs is especially striking during activation of sympathetic nerves, with nerve‐released norepinephrine causing constriction of cutaneous blood vessels by preferentially activating α2‐ARs (Flavahan, 2015a; Flavahan et al., 1984, 1987). In contrast to α2‐ARs, local cooling inhibits constriction to other contractile stimuli including α1‐ARs (Flavahan et al., 1985; Flavahan & Flavahan, 2020). Indeed, if cutaneous sympathetic neurotransmission were dependent on smooth muscle α1‐ARs, as occurs in other vascular systems, then local cooling would likely inhibit rather than augment the resulting sympathetic constriction. In clinical settings, cooling‐induced dilatation is markedly increased when cold‐induced constriction is inhibited, e.g., by α2‐AR antagonism (Johnson et al., 2005; Pergola et al., 1993). It is normally observed as transient increases in blood flow during or as active dilatation on cessation of cold‐induced constriction (Flavahan & Flavahan, 2020). Cold‐induced dilatation likely acts to restrain cold‐induced constriction and prevent tissue injury (Flavahan & Flavahan, 2020). Indeed, this response appears to be impaired in Raynaud's phenomenon, which may contribute to the increased magnitude and delayed recovery of cold‐induced constriction in this condition and the resulting tissue injury (Flavahan & Flavahan, 2020).

In isolated cutaneous arteries, cooling‐induced dilatation is mediated by increased communication through myoendothelial gap junctions (MEGJs) and amplification of endothelium‐derived hyperpolarization‐type (EDH‐type) dilatation (Flavahan & Flavahan, 2020). MEGJs are located on myoendothelial projections, which are physical connections between endothelial and smooth muscle cells through holes in the internal elastic lamina (IEL) (Garland & Dora, 2017; Sandow et al., 2009). These junctions enable the bidirectional movement of ions and signaling mediators. EDH is initiated by activation of endothelial calcium‐activated potassium channels (K_Ca_) of small (SK3) and intermediate (IK1) conductance (Garland & Dora, 2017). Hyperpolarization can then be conducted through MEGJs to the smooth muscle, resulting in dilatation. The endothelial K_Ca_ channels can be activated by discrete elevations in Ca^2+^ following endothelial stimulation by, e.g., acetylcholine, or following movement of muscle‐derived mediators (IP_3_, Ca^2+^) through MEGJs (Garland & Dora, 2017; Ottolini et al., 2019; Sandow et al., 2009). This latter response is termed myoendothelial feedback. The cooling‐induced increase in myendothelial communication amplified both EDH‐type responses, increasing dilatations to acetylcholine and depressing constriction to U46619 (Flavahan & Flavahan, 2020). In each case, the dilatation was resistant to inhibition of NO and prostacyclin production but was inhibited by combined blockade of SK3 and IK1, the original defining pharmacological profile of EDH dilatation (Flavahan & Flavahan, 2020; Garland & Dora, 2017).

Our understanding of the roles of endothelial SK3 and IK1 is now more refined. IK1 has been localized consistently within myoendothelial projections, emphasizing their importance to EDH and their apparent singular role in mediating myoendothelial feedback (Garland & Dora, 2017, 2021; Kerr et al., 2012; Murphy & Sandow, 2019). In contrast, SK3 has been localized primarily to the endothelial surface with increased localization at inter‐endothelial junctions (Garland & Dora, 2017, 2021; Kerr et al., 2012; Murphy & Sandow, 2019). SK3 is therefore considered to have reduced impact on EDH mechanisms and does not appear to contribute to myoendothelial feedback (Garland & Dora, 2017, 2021; Kerr et al., 2012; Murphy & Sandow, 2019). The aim of the present study was to determine whether specialization in the cutaneous circulation extends to EDH mechanisms and specifically to assess the individual involvement of IK1 and SK3 channels in cooling‐induced cutaneous dilatation. Experiments were conducted in isolated cutaneous mice tail arteries, a model of the human acral digital circulation (Chotani et al., 2000; Flavahan & Flavahan, 2020). The functional role of these K_Ca_ channels in cooling‐induced cutaneous dilatation as well as their expression and localization were investigated. The results suggest that cooling‐induced dilatation is mediated solely by SK3 channels that are localized to myoendothelial projections and that IK1 channels are neither expressed nor functional in these cutaneous arteries. This unusual specialization may enable EDH‐type mechanisms to be more active at cooler temperatures and so contribute to cooling‐induced dilatation.

## 2 METHODS

### 2.1 Animal care

Male C57BL6 mice (3–4 months) were obtained from the Jackson Laboratory, housed in institutional facilities, and fed rodent chow. Animal use was approved by the IACUC and complied with NIH guidelines. Mice were euthanized by CO_2_ asphyxiation, and tail arteries were removed and placed in cold Krebs–Ringer bicarbonate solution (control solution) (Flavahan & Flavahan, 2020).

### 2.2 Functional responses

Adjacent arterial segments were studied in parallel and randomly assigned to treatment groups, with one segment remaining untreated (Flavahan & Flavahan, 2020). Tail arteries were cannulated with micropipettes, secured within a microvascular chamber, and maintained at a transmural pressure of 60 mmHg (Living Systems) (Flavahan & Flavahan, 2020). Arteries were superfused with control solution (37°C, pH 7.4, 16% O_2_, 5% CO_2_, balance N_2_), and the chamber was placed on the stage of an inverted microscope. Arterial diameter and pressure were continuously monitored and recorded (BIOPAC). Cumulative concentration‐effect curves were generated by increasing the agonist concentration once the response to the previous concentration had stabilized. Constriction to U46619 was evaluated under basal conditions, and dilatation to acetylcholine was assessed during stable constriction to U46619 (Flavahan & Flavahan, 2020). In these arteries, acetylcholine evokes endothelium‐dependent dilatation with no significant effect on smooth muscle (Flavahan & Flavahan, 2020).

Effects of cooling were analyzed by reducing the superfusate temperature from 37°C to 28°C for 30 min prior to constricting the arteries with U46619 and maintained at that temperature (Flavahan & Flavahan, 2020). 28°C represents a moderate level of local cooling and causes a powerful increase in α2‐adrenergic constriction, the primary mechanism underlying cold‐induced constriction (Flavahan & Flavahan, 2020). Moreover, 30 min provides sufficient time for the effect of cooling to stabilize (Flavahan & Flavahan, 2020). All experiments were performed in the presence of indomethacin (10 μM) plus N^G^‐nitro‐L‐arginine methyl ester (LNAME, 100 μM) to inhibit the production of prostaglandins and NO, respectively (Flavahan & Flavahan, 2020). When assessing the effects of antagonists on constrictor or dilator responses, they were administered 60 min prior to U46619 and remained in contact with the arteries while assessing constrictor and dilator responses (Flavahan & Flavahan, 2020).

As reported previously, arteries displayed minimal to no basal contractile tone, and there was no significant effect of cooling and/or the inhibitors on baseline diameter (Flavahan & Flavahan, 2020). With UCL1684, basal diameters at 37°C were 375.8 ± 7.6 μ and 371.1 ± 8.1 μ, and at 28°C they were 366.4 ± 10.1 μ and 363.5 ± 12.7 μ for control and UCL1684‐treated arteries, respectively (*n* = 11, *p* = NS for all comparisons). Likewise, with TRAM34, diameters at 37°C were 358.8 ± 8.1 μ and 343.4 ± 10.7 μ, and at 28°C, they were 358.8 ± 8.3 μ and 362.4 ± 8.3 μ for control and TRAM34‐treated arteries, respectively (*n* = 13, *p* = NS for all comparisons).

### 2.3 Immunofluorescence

Tail arteries were mounted in specialized chambers that enabled arteries to be rapidly transferred from control solution to paraformaldehyde (3%, room temperature or RT, 20 min). Arteries were opened longitudinally during fixation, rinsed in PBS (3 × 10 min), then in blocking buffer (donkey serum 1%, glycine 20 mM, and Tween 20 0.2% in PBS), and incubated overnight in blocking buffer (RT) with a goat polyclonal antibody to VE‐cadherin (clone C‐19, 1:500, catalog no. SC6458; Santa Cruz Biotechnology) and either rabbit polyclonal antibody to SK3 (1:100, catalog no. APC‐025; Alomone) or a rabbit polyclonal antibody to IK1 (1:100, catalog no. APC‐064; Alomone). In parallel analyses, arteries were also treated with SK3 or IK1 antibodies after being pre‐adsorbed with blocking peptides (catalog nos. BLP‐PC025, BLP‐PC064; Alomone), according to manufacturer's guidelines. Arteries were then rinsed in PBS (3 × 10 min) and incubated (2 h) with AlexaFluor488‐labeled donkey anti‐rabbit antibody (1:200, catalog no. 711‐545‐152; Jackson ImmunoResearch), rhodamine‐labeled donkey anti‐goat antibody (1:200, catalog no. 705‐025‐147; Jackson ImmunoResearch), and Alexa633 hydrazide (0.05 μM) (catalog no. A30634; ThermoFisher). Samples were viewed using a Leica SP5 laser scanning microscope. Images (1024 × 1024 pixels) were obtained with a 63× objective (1.4 NA), using sequential acquisition, a pinhole of two Airy units, scan speed of 400 Hz, 6‐line averaging, optical zoom of 3.0, and excitation/emission settings for AlexaFluor488 (488 nm/497‐541 nm) Rhodamine (543 nm/565‐624 nm) and AlexaFluor633 (633 nm/641‐707 nm). Images were obtained and processed using the same hardware and software settings.

### 2.4 Western blots

Each artery was placed in 50 μL modified RIPA buffer (50 mM Tris pH 7.6, 150 mM NaCl, 0.5% Triton X‐100, 0.5% deoxycholate, 1% SDS, 2 mM EDTA, 20 μg/mL leupeptin, 20 μg/mL aprotinin, 10 mM NaF, 0.5 mM 4‐benenesulfonyl fluoride hydrochloride, 1 mM sodium orthovanadate) then vortexed (5 s) and boiled (5 min) before being sonicated (10 s) then boiled and vortexed again. Samples were centrifuged (16,000 *g*, 10 min) and the supernatant harvested for analysis. 15 μg of arterial protein lysates were processed for SDS‐PAGE, with 15 μg of HUVEC lysates included as a positive control. For Western blot analysis, membranes were blocked with 1% BSA in TBST buffer (3 h, RT) then incubated with primary antibodies, IK1 (1:1000, catalog no. APC‐06; 4Alomone Lab), SK3 (1:1000, catalog no. P4743; Millipore) or β‐actin (1:3000, catalog no. SC47778; Santa Cruz Biotechnology) (4°C,18 h). Membranes were washed with TBST (3 × 10 min) before being incubated with peroxidase‐conjugated goat anti‐rabbit IgG or goat anti‐mouse IgG (Jackson ImmunoResearch Laboratories, catalog nos. 111‐035‐003 and 115‐035‐003, respectively) (1 h, RT) followed by TBST washes (3 × 10 min). Blots were then developed using enhanced chemiluminescence (Thermo Fisher).

### 2.5 Drugs

Acetylcholine (catalog no. A9101), indomethacin (catalog no. I7378), and LNAME (catalog no. N5751) were from Sigma–Aldrich; U46619 (catalog no. 1932), UCL1684 (catalog no. 1310), and TRAM34 (catalog no. 2946) were from Tocris.

### 2.6 Data analysis

Functional responses were expressed as a percent change in baseline diameter. For acetylcholine, agonist concentrations causing 50% dilatation of constriction to U46619 (EC_50_) were determined by regression analysis and compared as log EC_50_ (Flavahan & Flavahan, 2020). Maximum responses were determined as the maximal observed dilatation of the constriction to U46619 (Flavahan & Flavahan, 2020). When assessing constrictor responses to U46619, concentrations causing 20% constriction from baseline diameter (EC_20_) were determined by regression analysis and compared as log EC_20_ (Flavahan & Flavahan, 2020). Constriction was constrained to prevent endothelial injury, and so maximum constrictions were not determined (Flavahan & Flavahan, 2020). Unless stated otherwise, data is expressed as means ± SEM for n number of experiments and number of animals from which arteries were taken. SEM rather than SD was used to prevent excessive overlapping of error bars on line graphs and so facilitate interpretation of the data. Statistical evaluation of the data was performed with GraphPad Prism 9 software, using Student's *t*‐test for paired or unpaired observations. When more than two means were compared, one‐way or two‐way repeated measures ANOVA was used. If a significant F value was found, then the Tukey–Kramer test for multiple comparisons was employed to identify differences among groups. Values were considered to be statistically different when *p* < 0.05.

## 3 RESULTS

### 3.1 K_Ca_ function

**FIGURE 1:**
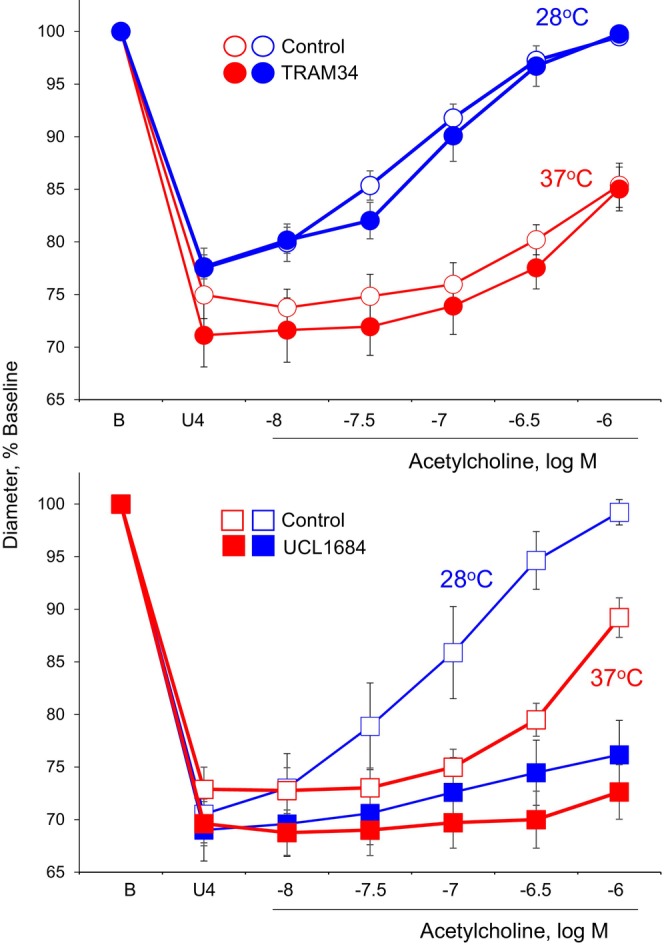
Effects of inhibiting IK1 (TRAM34, 5 μM) (top) or SK3 channels (UCL1684, 5 μM) (bottom) on dilatation of mice tail arteries to acetylcholine at 37°C and 28°C. Responses were analyzed during stable constriction to U46619 (U4) in the presence of indomethacin (10 μM) plus LNAME (100 μM). Data are expressed relative to the baseline diameter (B) and presented as means ± SEM (*n* = 5 top, *n* = 6 bottom). See Table 1a for statistical analyses.

As reported previously (Flavahan & Flavahan, 2020), during inhibition of NO and prostacyclin production, cooling to 28°C increased the remaining EDH‐type component of dilatation to acetylcholine, causing a significant increase in the maximal dilatation and a 14‐fold leftward shift in the concentration‐effect curve to the agonist (log shift of 1.15 ± 0.14, −fold, *n* = 11) (Figure 1, Table 1). Combined inhibition of SK3 (UCL1684) and IK1 (TRAM34) markedly depressed these dilatations (Flavahan & Flavahan, 2020). In the present study, IK1 inhibition alone (TRAM34, 5 μM) had no significant effect on the concentration‐effect curve to acetylcholine at either temperature, neither significantly affecting the maximal response nor the log EC_50_ to the agonist at 37°C or at 28°C. (Figure 1, Table 1). By contrast, SK3 inhibition alone (UCL1684, 5 μM) significantly depressed the concentration‐effect curves to acetylcholine, markedly inhibiting the maximal dilatation at 37°C and 28°C (Figure 1, Table 1). The residual response to acetylcholine after SK3 inhibition was too small to determine log EC_50_ values (Figure 1, Table 1a). Therefore, SK3 inhibition alone mimicked the combined effects of SK3 plus IK1 inhibition, whereas IK1 inhibition alone was without significant effect.

**TABLE 1:** Role of K_Ca_ in vasomotor responses of mice tail arteries.

A: Dilatation to acetylcholine		
Treatment^a^	Maximum^b^ ^,^ ^c^	Log EC_50_ ^c^ ^,^ ^d^
Control 37°C (6)	80.3 ± 4.4%	−6.12 ± 0.07
UCL1684 37°C (6)	19.1 ± 5.6%***	ND
Control 28°C (6)	98.9 ± 3.8%^@^	−7.12 ± 0.16^@@@^
UCL1684 28°C (6)	24.2 ± 4.6%***	ND
Control 37°C (5)	70.7 ± 6.3%	−5.87 ± 0.13
TRAM34 37°C (5)	69.3 ± 7.6%	−5.89 ± 0.12
Control 28°C (5)	98.0 ± 3.0%^@^	−7.21 ± 0.08^@@@^
TRAM34 28°C (5)	99.6 ± 3.3%^@^	−7.04 ± 0.09^@@@^

B: Constriction to U46619	
Treatment^a^	Log EC_20_ ^c^
Control 37°C (7)	−8.38 ± 0.04
Control 28°C (7)	−7.78 ± 0.01^@@@^
UCL1684 37°C (7)	−8.35 ± 0.02
UCL1684 28°C (7)	−8.33 ± 0.03***
Control 37°C (6)	−8.22 ± 0.05
Control 28°C (6)	−7.75 ± 0.03^@@@^
TRAM34 37°C (6)	−8.20 ± 0.06
TRAM34 28°C (6)	−7.76 ± 0.04^@@@^

^**a**^ Number of experiments indicated in parentheses. All experiments performed in the presence of indomethacin (10 μM) plus LNAME (100 μM).

^**b**^ Refers to maximal‐observed dilatation.

^**c**^ Symbols: *, refers to statistically significant difference compared to matched control group. ^@^, refers to statistically significant difference compared to corresponding value at 37°C. For each: one symbol, *p* < 0.05; three symbols, *p* < 0.001.

^**d**^ ND, EC_50_ values could not be determined.

**FIGURE 2:**
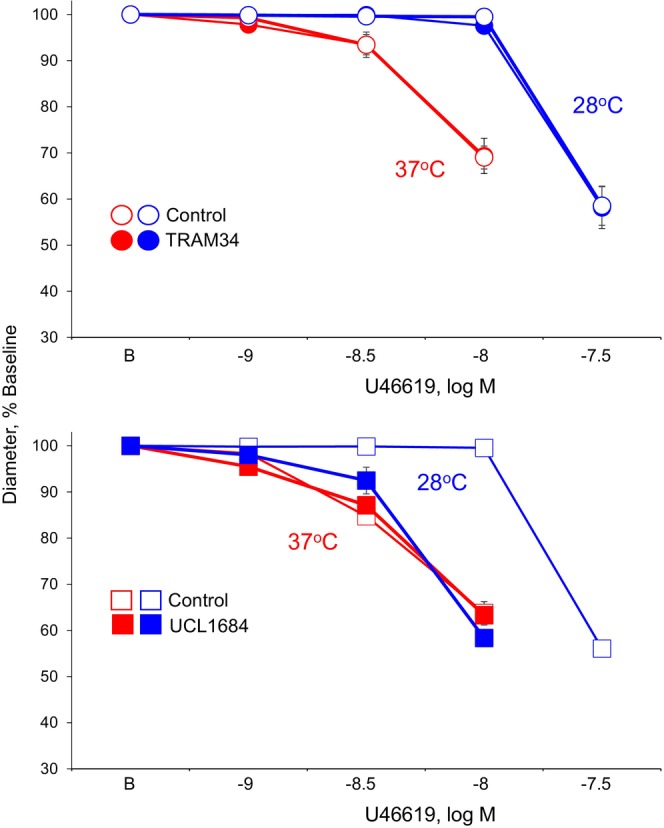
Effects of inhibiting IK1 (TRAM34, 5 μM, top) or SK3 channels (UCL1684, 5 μM, bottom) on constriction of mice tail arteries to U46619 at 37°C and 28°C. Responses were analyzed under baseline conditions in the presence of indomethacin (10 μM) plus LNAME (100 μM). Data are expressed relative to the baseline diameter (B) and presented as means ± SEM (*n* = 6 top, *n* = 7 bottom). Note that some symbols are obscured because of overlapping responses. See Table 1b for statistical analyses.

Cooling to 28°C inhibited constriction to U46619 causing a significant 3.5‐fold rightward shift in the concentration‐effect curve (log shift of 0.54 ± 0.04, *n* = 13) (Figure 2, Table 1). As reported previously, combined inhibition of SK3 (UCL1684) and IK1 (TRAM34) did not affect constriction to U46619 at 37°C, but increased the constriction to the agonist at 28°C and abolished the inhibitory effects of cooling (Flavahan & Flavahan, 2020). In the present study, IK1 inhibition alone (TRAM34, 5 μM) did not significantly affect constriction to U46619 at 37°C or at 28°C (Figure 2, Table 1). By contrast, SK3 inhibition alone (UCL1684, 5 μM) did not significantly affect constriction to U46619 at 37°C, but increased constriction to the agonist at 28°C, causing a significant 3.6‐fold leftward shift in the concentration‐effect curve (log shift of 0.55 ± 0.03, *n* = 7), thereby abolishing the inhibitory effect of cooling (Figure 2, Table 1). Therefore, SK3 inhibition alone mimicked the combined effects of SK3 plus IK1 inhibition, whereas IK1 inhibition alone was without significant effect.

### 3.2 K_Ca_ protein expression and localization

**FIGURE 3:**
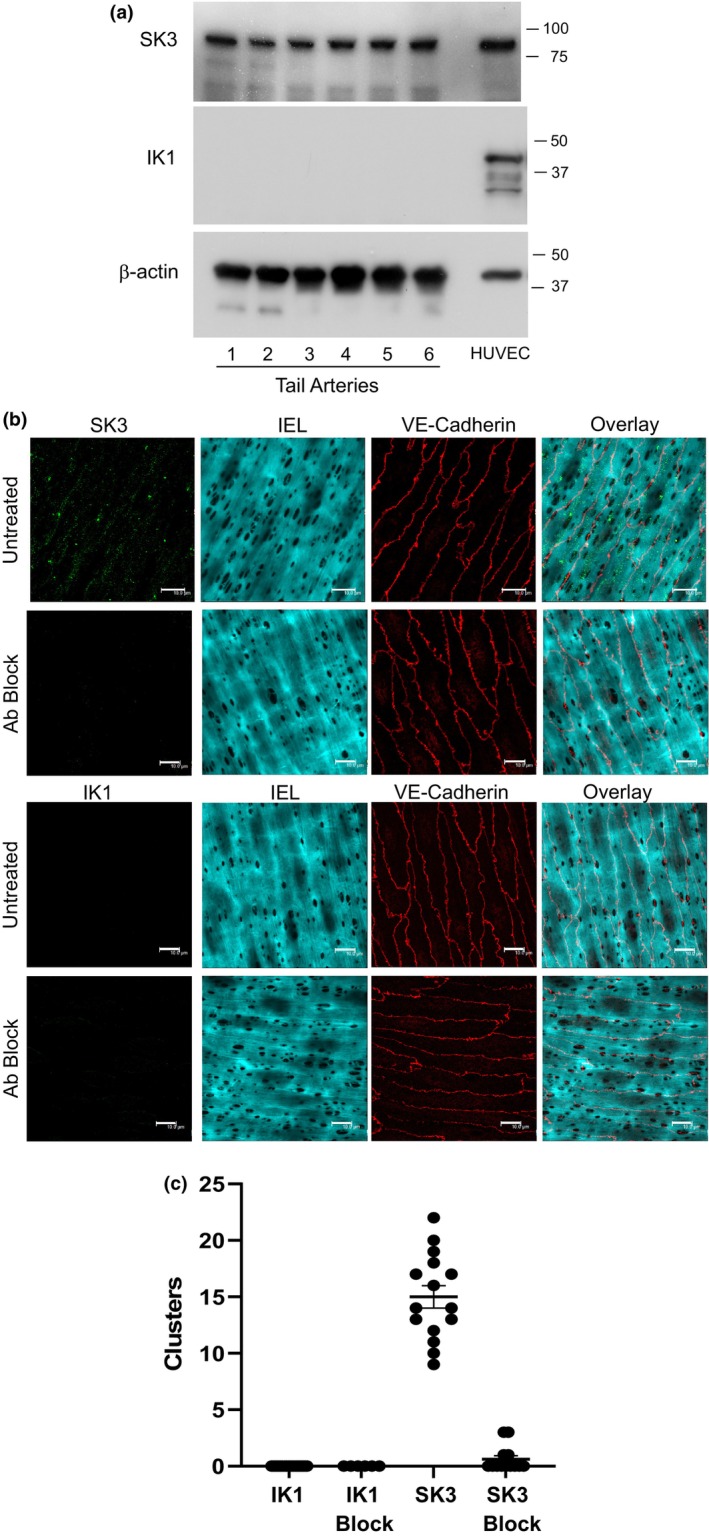
Analysis of K_Ca_ channel expression in mice tail arteries. (a) Immunoblot analysis. Lysates from six tail arteries from different mice were assessed, and HUVECs were included as a positive control. Samples were assessed for β‐actin to confirm adequate sample loading. (b) Immunofluorescent analysis of K_Ca_ channels. Staining with SK3 and IK antibodies was evaluated under control conditions (untreated) and after pre‐adsorption with blocking peptides (Ab Block). Staining for VE‐cadherin and IEL was included to identify endothelial cells and potential location of myoendothelial projections. Line represents 10 μ. (c) Grouped data for immunofluorescence. The number of clusters associated with holes in the IEL was quantified for each field of view (an area of 82 μ squared), and data is presented as means ± SEM for 6–20 fields of view from *n* = 4 arteries from different mice.

Immunoblot analyses demonstrated significant SK3 expression but failed to detect IK1 expression in mice tail arteries (Figure 3a). In immunofluorescent analyses, specific SK3 staining was localized to punctate staining associated with holes in the internal elastic lamina, consistent with localization to myoendothelial projections (Figure 3b, c). IK1 was not detected by immunofluorescence in mice tail arteries (Figure 3b, c).

## 4 DISCUSSION

The cutaneous vascular system has unique characteristics that optimize its role in thermoregulation (Flavahan, 2015a). For example, smooth muscle α2‐ARs, the main cellular effectors for cold‐induced vasoconstriction, are much more prominent in cutaneous compared to other vascular systems (Chotani et al., 2004; Flavahan, 2015a). The present study extends this specialization to cold‐induced vasodilatation. The results suggest that, unlike other vascular systems, EDH‐type dilatations in the cutaneous system are dependent exclusively on SK3 with no contribution from IK1. This distinct specialization may enable EDH‐type dilatations to be amplified by cooling and so contribute to cooling‐induced cutaneous dilatation.

The IK1 protein was not detected in mouse tail arteries by immunoblot or immunofluorescent analyses. The immunoblot approach detected the expected ~45kD protein in cultured HUVECs (Figure 3) and in mice brain lysates (data not shown). Likewise, immunofluorescent analyses, which employed a well‐characterized antibody and approach that detected IK1 in non‐cutaneous arteries (Dora et al., 2008; Potocnik et al., 2009; Sandow et al., 2006), did not detect IK1 in mouse tail arteries. By contrast, abundant expression of SK3 was observed by immunoblot analyses of cutaneous arterial lysates. Moreover, SK3 was localized by immunofluorescence to punctate staining associated with holes in the IEL, consistent with localization to myoendothelial projections. The presence of SK3 but not IK1 is consistent with the functional analysis of EDH‐type dilatations in these arteries. IK1 inhibition had no effect on EDH‐type dilatations to acetylcholine at 37°C or the amplified dilatations at 28°C, or on the myoendothelial feedback of U46619 constriction that was uncovered by cooling. In contrast, these responses were markedly reduced or abolished by SK3 inhibition. Indeed, SK3 inhibition had a similar inhibitory effect to that produced by combined inhibition of IK1 and SK3 channels, observed previously (Flavahan & Flavahan, 2020).

EDH‐type dilatations have been studied most intensively in rat mesenteric arteries. These arteries express SK3 and IK1 (Khaddaj‐Mallat et al., 2018; Schach et al., 2014): IK1 is localized to punctate staining near holes in the IEL, consistent with myoendothelial projections, whereas SK3 is localized to inter‐endothelial junctions and is generally excluded from myoendothelial projections (Dora et al., 2008; Haddock et al., 2011; Sandow et al., 2006). In rat mesenteric arteries, IK1 and SK3 both contribute to EDH‐type dilatation evoked by acetylcholine (Dora et al., 2008; Haddock et al., 2011; Kloza et al., 2017; Yarova et al., 2013), whereas only IK1 channels contribute to myoendothelial feedback (Wei et al., 2018). This is thought to reflect the preferential localization of IK1 at myendothelial projections, enabling them to be activated by smooth muscle‐derived mediators (Ca^2+^, IP_3_) following transit through MEGJs (Wei et al., 2018). Exclusive localization of IK1 at myoendothelial projections is also observed in hamster retractor muscle feed arteries and human mesenteric arteries (Chadha et al., 2011; Tran et al., 2012), whereas both IK1 and SK3 have been localized to these structures in mice cremaster muscle arterioles and mesenteric arteries (Ottolini et al., 2020; Potocnik et al., 2009). However, even in these latter arteries, myoendothelial feedback is mediated exclusively by IK1 (Nausch et al., 2012). Indeed, the consensus is that IK1 and SK3 channels can both contribute to EDH‐type dilation initiated by endothelial activation, but that IK1 channels are exclusively involved in mediating myoendothelial feedback (Garland & Dora, 2017; Kerr et al., 2012, 2015; Sandow et al., 2009; Tran et al., 2012). The exclusive role of SK3 channels in mediating EDH‐type dilatations in cutaneous arteries is therefore highly unusual and likely reflects unique adaptation or specialization of the cutaneous vascular system to enable EDH‐type dilatation to be augmented by cooling.

Compared to other small arteries, including rat mesenteric arteries, EDH‐type dilatation in cutaneous arteries at 37°C is a minor contributor to dilatation. At high concentrations, acetylcholine can barely achieve 50% dilatation of constriction to U46619. Likewise, there is no evidence of myoendothelial feedback at this temperature because inhibition of endothelial K_Ca_ does not increase constriction to U446619. This minimal activity of EDH‐type activity likely reflects the absence of IK expression and the poor communication through MEGJs at this temperature (Flavahan & Flavahan, 2020). However, this minimal activity is rescued by cooling, enabling EDH‐type dilatation to acetylcholine to achieve 100% dilatation and revealing myoendothelial feedback during smooth muscle constriction.

Endothelial hyperpolarization resulting from activation of endothelial K_Ca_ channels can be transmitted to smooth muscle cells via accumulation of K^+^ in the intercellular space between the two cell types (“K cloud”), which can activate smooth muscle Na^+^‐K^+^‐ATPase and inwardly rectifying potassium channels (K_IR_), and/or via direct coupling through myoendothelial gap junctions (MEGJs). In cutaneous arteries, cooling abolished the dilatation evoked by increasing K^+^ (5 mM), which mimics the activity of a K cloud (Flavahan & Flavahan, 2020). This likely reflects an inhibitory effect of cooling on Na^+^‐K^+^‐ATPase (Flavahan & Flavahan, 2020). By contrast, cooling increased dye transfer between smooth muscle and endothelial cells, and the cooling‐induced increase in EDH‐type dilatation was prevented by blocking gap junctions (Flavahan & Flavahan, 2020). Therefore, the results suggest that the cooling‐induced amplification of SK3‐mediated dilatation is mediated solely by MEGJs, with no contribution from an extracellular K cloud. In rat mesenteric arteries, the EDH‐type dilatations are mediated by both direct coupling through MEGJs and the dilator activity of a K cloud (Dora et al., 2008; Harno et al., 2008). Importantly, the activity of IK1 channels is preferentially coupled to Na^+^‐K^+^‐ATPase, whereas SK3 activity is preferentially coupled to MEGJs (Dora et al., 2008; Harno et al., 2008). The preferential coupling of SK3 channels to MEGJs and the inhibitory effect of cooling on dilatation to extracellular K may provide a mechanistic basis for the sole reliance of cutaneous arteries on SK3.

NO synthase is localized to myoendothelial projections and can be stimulated by localized signaling within this specialized domain, including during myoendothelial feedback (Dora et al., 1997; Mutchler & Straub, 2015). Likewise, activation of IK1 and SK3 can augment NO‐mediated dilatation (Kerr et al., 2015; Sheng et al., 2009). However, hemoglobin α (HBα) is also localized to these projections and acts to scavenge NO (Mutchler & Straub, 2015). Indeed, the increased prominence of EDH in the microcirculation may reflect an increased scavenging role of HBα (Mutchler & Straub, 2015). After inhibiting NO synthase, moderate cooling markedly increased the remaining EDH‐type dilatation to acetylcholine in mice tail arteries (Flavahan & Flavahan, 2020). By contrast, after inhibiting EDH‐type dilatation, moderate cooling minimally affected the remaining NO‐dependent dilatation to acetylcholine (Flavahan & Flavahan, 2020). This suggests that cooling exerts a dilator influence by selectively increasing EDH‐type dilatation without significantly impacting endothelial NO activity (Flavahan & Flavahan, 2020). Therefore, the pathways responsible for the acetylcholine‐induced stimulation of NO synthase, which might include activation of SK3, is not influenced by cooling. The effect of cooling to inhibit constriction to U46619 was not affected by inhibition of NO synthase suggesting that, at least for myoendothelial feedback, SK activity is preferentially linked to EDH‐type dilatation rather than NO‐mediated dilatation (Flavahan & Flavahan, 2020).

In rat mesenteric arteries, thromboxane receptor stimulation by U46619 selectively inhibited the component of EDH and dilatation to acetylcholine that was mediated by SK3 channels without suppressing the component mediated by IK1 channels (Crane & Garland, 2004). This inhibitory effect did not occur in response to activation of α1‐AR by phenylephrine. The underlying mechanism remains undefined, although the authors assume it reflected activation of endothelial thromboxane receptors (Crane & Garland, 2004). This observation does not have relevance for the results of the present study. The inhibitory effect of U46619 on SK3 was a slowly developing phenomenon and was only observed following multiple sequential exposures to the thromboxane mimic (Crane & Garland, 2004). In the present study, arteries were only exposed to U46619 once, which was insufficient to inhibit SK3 activity in rat mesenteric arteries (Crane & Garland, 2004).

**FIGURE 4:**
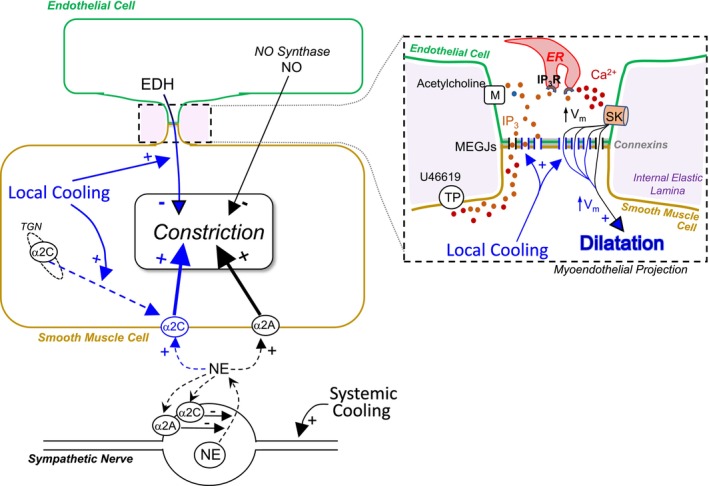
Schematic representation of the effects of cooling on vasomotor responses in acral cutaneous blood vessels. Under thermoneutral conditions and during elevated activity in response to mild or moderate cooling, sympathetic adrenergic nerves release norepinephrine (NE) that initiates constriction by activating α_2A_‐ARs located on smooth muscle cells. Local skin cooling amplifies sympathetic vasoconstriction by stimulating the translocation of α2C‐ARs from the *trans*Golgi network (TGN) to the surface of smooth muscle cells, where they are activated by NE. Endothelial cells can exert a powerful dilator influence through the production of NO (from endothelial NO synthase, eNOS). Local cooling amplifies EDH‐type dilatation by increasing direct communication between endothelial and smooth muscle cells through MEGJs located within myoendothelial projections. ***Inset*** represents an expanded view of a myoendothelial projection, where endothelial and smooth muscle cells make contact through holes in the internal elastic lamina. Localized signaling and expression of mediators in myoendothelial projections contribute to EDH. Activation of endothelial SK3 channels within myoendothelial projections generate endothelial hyperpolarization (increased Vm, membrane potential) that is transmitted through MEGJs to cause smooth muscle hyperpolarization and dilatation. The endothelial K_Ca_ channels can be activated by discrete elevations in Ca^2+^ associated with endothelial stimulation (e.g., by acetylcholine ACH) or following diffusion of IP_3_ from activated smooth muscle cells through MEGJs (e.g., by U46619) resulting in activation of IP_3_ receptors (IP_3_Rs) on endothelial endoplasmic reticulum (ER) and release of ER Ca^2+^ (myoendothelial feedback). At warm temperatures, there is limited conduction through MEGJs and therefore diminished EDH‐type dilatations. Moderate local cooling markedly increases myoendothelial communication through MEGJs and enables endothelial hyperpolarization to be efficiently transferred to the smooth muscle cells resulting in marked EDH‐type dilatation. Blue arrows and structures represent processes activated by local cooling.

The results of the present study provide for a more complete understanding of the response of the acral cutaneous circulation to cold exposure (Figure 4). Under thermoneutral conditions, sympathetic adrenergic nerves release norepinephrine to cause constriction by activating α_2A_‐ARs on smooth muscle cells (Chotani et al., 2000; Flavahan, 2015a). This sympathetic activity will increase in response to mild to moderate systemic cooling to restrict heat loss (Chotani et al., 2000; Flavahan, 2015a). In response to moderate local skin cooling, α2C‐ARs translocate from the *trans*Golgi network (TGN) to the surface of smooth muscle cells, where they are activated by norepinephrine and are responsible for local cooling‐induced augmentation of the sympathetic constriction (Bailey et al., 2004, 2005; Jeyaraj et al., 2001). However, local cooling also initiates a protective mechanism to cause dilatation that restrains cold‐induced constriction and prevents ischemic tissue injury (Flavahan & Flavahan, 2020). Indeed, this dilator influence is reportedly reduced in Raynaud's phenomenon and may contribute to tissue injury in this condition (Flavahan & Flavahan, 2020). This important dilator response reflects an increase in EDH‐type dilatation mediated by endothelial SK3 potassium channels and an increase in direct myoendothelial communication.

### 4.1 Limitations

We lack the technical ability to measure membrane potential or ion channel currents in endothelial or smooth muscle cells of small arteries. Therefore, although our functional responses are consistent with those mediated by EDH, we refer to them as EDH‐type rather than EDH. This is standard practice in this research area. An additional limitation is that experiments were performed only in male mice. Cold‐induced vasoconstriction is increased in women compared to men, which likely reflects an increased sensitivity of the female cutaneous circulation to sympathetic vasoconstriction and an effect of estrogen to increase expression of cold‐sensitive α2C‐ARs (Eid et al., 2007; Flavahan, 2015a, 2015b). Indeed, this explains why women are much more likely to suffer from Raynaud's phenomenon and why symptoms improve after menopause, but not in postmenopausal women taking estrogen replacement therapy (Greenfield et al., 2022). Interestingly, EDH is reported to be increased in females compared to males, which may reflect a positive effect of estrogen on endothelial K_Ca_ or MEGJs (Leung & Vanhoutte, 2017). Therefore, in addition to amplifying mechanisms underlying cold‐induced cutaneous constriction, estrogen may also amplify cold‐induced dilatation, which would act to restrain the constriction and prevent tissue injury.

## AUTHOR CONTRIBUTIONS

Nicholas Flavahan designed, performed, analyzed and interpreted experiments, and drafted the manuscript. Fumin Chang designed, performed, analyzed and interpreted experiments, and edited the manuscript. Sheila Flavahan designed, performed, analyzed and interpreted experiments, and edited the manuscript.

## FUNDING INFORMATION

This study was funded by the American Heart Association (TPA35490253).

## CONFLICT OF INTEREST STATEMENT

All authors declare no conflict of interest.

## ETHICS STATEMENT

All animal experiments followed the NIH guidelines for care and use of laboratory animals and were approved by the Institutional Animal Care and Use Committee of the Johns Hopkins University School of Medicine.

## Data Availability

The data supporting this study are available upon request.
